# Ultrastructural and Molecular Analysis of Vascular Smooth Muscle Cells During the Switch from a Physiological to a Pathological Phenotype

**DOI:** 10.3390/biomedicines13051127

**Published:** 2025-05-06

**Authors:** Elisa Persiani, Elisa Ceccherini, Alessandra Falleni, Ilaria Gisone, Chiara Ippolito, Letizia Mattii, Antonella Cecchettini, Federico Vozzi

**Affiliations:** 1 Institute of Clinical Physiology, National Research Council, 56124 Pisa, Italy; elisapersiani@cnr.it (E.P.); ilariagisone@cnr.it (I.G.); antonella.cecchettini@unipi.it (A.C.); federico.vozzi@cnr.it (F.V.); 2 Department of Clinical and Experimental Medicine, University of Pisa, 56126 Pisa, Italy; alessandra.falleni@unipi.it (A.F.); chiara.ippolito@unipi.it (C.I.); letizia.mattii@unipi.it (L.M.)

**Keywords:** vascular smooth muscle cells, phenotype switching, morphology, vascular calcification, atherosclerosis, autophagy

## Abstract

**Background/Objectives**: Under physiological conditions, vascular smooth muscle cells (VSMCs) are in a quiescent contractile state, but under pathological conditions, such as atherosclerosis, they change their phenotype to synthetic, characterized by increased proliferation, migration, and production of an extracellular matrix. Furthermore, VSMCs can undergo calcification, switching to an osteoblast-like phenotype, contributing to plaque instability. **Methods**: In this study, we analyzed the phenotypic changes in VSMCs during the transition from a physiological to a pathological state, a key process in the progression of atherosclerosis, using confocal and transmission electron microscopy, real-time PCR, and intracellular calcium quantification. **Results**: Confocal and transmission electron microscopy revealed a prominent remodeling of the actin cytoskeleton, increasing autophagic vacuoles in synthetic VSMCs and the deposition of calcium microcrystals in calcified cells. Immunofluorescence analysis revealed differential expression of α-SMA (contractile marker) and galectin-3 (synthetic marker), confirming the phenotypic changes. Real-time PCR further validated these changes, showing upregulation of RUNX-2, a marker of osteogenic transition, in calcified VSMCs. **Conclusions**: This study highlights the dynamic plasticity of VSMCs and their role in atherosclerosis progression. Understanding the characteristics of these phenotypic transitions can help develop targeted therapies to mitigate vascular calcification and plaque instability, potentially countering cardiovascular disease.

## 1. Introduction

Atherosclerosis is a primary contributor to cardio-cerebrovascular ischemic diseases, significantly impacting global mortality rates. The most widely accepted theory regarding the pathophysiology of atherosclerosis is that it is a chronic inflammatory disease of the blood vessel wall, with key processes including lipid deposition, leukocyte infiltration, endothelial cell dysfunction, alterations in vascular smooth muscle cells (VSMCs), and extracellular matrix accumulation [1,2]. VSMCs display a remarkably high degree of plasticity and a number of different VSMC phenotypes have only partially been characterized [3]. Morphological and immunohistological analyses allowed the characterization of two principal phenotypes, which have historically been contractile and synthetic. In healthy conditions, VSMCs—the main cellular component of the artery wall—are quiescent and have an expanded nucleus and a fusiform shape [4,5]. Alpha-smooth muscle actin (α-SMA) and myosin isoforms, essential for controlling vascular tone, are abundant in the contractile fibers of these differentiated cells, which also show modest synthetic activity and a low proliferative propensity [5,6]. One term used to describe VSMCs in this distinct physiological state is “contractile”. When cultivated in vitro with bovine serum or in vivo under pathological conditions like atherosclerosis, VSMCs can become activated and change into a dedifferentiated “synthetic” phenotype, which is deemed pathological because it is linked to the advancement of cardiovascular disease [5,7,8]. The contractile apparatus of synthetic VSMCs is significantly reduced while their capacity to synthesize proteins increases. They acquire the capacity to proliferate, divide, and migrate, developing atherosclerotic plaques [6]. As their morphology changes, they lose the spindle shape and become enlarged with a fan-shaped appearance, featuring cell migration structures such as pseudopodia, lamellipodia, and filopodia [9,10]. Atherosclerosis is also associated with vascular calcification, which is characterized by the deposition of calcium crystals into the intima and media as microcalcifications, increasing the risk of plaque rupture [11,12]. Notably, calcified vascular lesions are associated with VSMC switching to osteoblast-like and chondrocyte-like cells [13]. This change is characterized by the loss of expression of contractile markers and the upregulation of chondrocyte- and osteoblast-like markers, such as the Runt-related transcription factor 2, osteopontin, osteocalcin, alkaline phosphatase, osterix, and collagen type II and X [14,15]. The presence of calcifying vesicles accompanies this process, the decreased expression of molecules that inhibit mineralization, and the production of a calcified extracellular matrix [16]. Additionally, galectin-3 plays a significant role in promoting VSMC calcification and is associated with the development of vulnerable plaques [17,18]. It induces a chronic inflammatory state at the cardiovascular level and is required for developing an osteoblast-like phenotype that encourages calcification [18,19]. Notably, it can be considered a marker of VSMCs undergoing phenotypic transitions in atherosclerotic lesions [20]. In this work, we used Human Coronary Artery Smooth Muscle Cells (HCASMCs) as an in vitro model to examine the phenotype switching at the ultrastructural level. Although the phenotypic change in these cells has been described and discussed extensively, a detailed ultrastructural description is lacking so far. Since its invention in the early 1930s, transmission electron microscopy (TEM) influenced the course of scientific research, providing new levels of magnification and resolution for exploring biological and non-biological samples [21,22]. Without a doubt, TEM is the gold standard for organelle characterization as it offers resolution that is orders of magnitude higher than any conventional light microscopy, allowing the display of membranes, cytoplasmic structures, luminal content, and structures within the cells [23]. Moreover, it is an indispensable tool to study normal and pathological characters at the subcellular level, as it is a powerful diagnostic approach for viral infection and provides the finest evaluation and detection of the severity of cell alteration [24]. It is also one of the best methods for detecting and localizing antigens (using immunolabeling techniques), especially when these are not detectable by other investigation techniques [25]. In the realm of cardiovascular disease in vitro models, this study aims to highlight the spatiotemporal regulation of the phenotypic modulation of VSMCs by investigating the different morphological properties classified into three categories: contractile, synthetic, and calcified. The micrographic analysis of cellular structure variations in VSMCs, corroborated by molecular analyses, may uncover fundamental mechanistic insights into phenotypic reprogramming.

## 2. Materials and Methods

### 2.1. Cell Culture and Maintenance

Human Coronary Artery Smooth Muscle Cells (HCASMCs, hereinafter referred to as VSMCs) were obtained from Thermo Fisher (Waltham, MA, USA). VSMCs were cultured in 25 cm^2^ flasks using DMEM high glucose medium (Merck, Darmstadt, Germany) supplemented with 10% FBS (Merck, Darmstadt, Germany) and a penicillin/streptomycin solution (Merck, Darmstadt, Germany, 100 I.U/mL and 100 μg/mL final concentration, respectively). The cells were maintained at 37 °C in a humidified atmosphere with 5% CO_2_. The medium was changed daily, and trypsinised cells were passaged upon reaching 90% confluence. VSMCs were subcultured for a maximum of four times and cells treated for TEM and confocal analyses were at the second passage. VSMCs were cultured in medium deprived of serum for at least 3 days to reproduce a contractile and quiescent phenotype (hereinafter referred to as “CON”). When the cells were cultured in a medium with FBS, they acquired the ability to migrate and proliferate [26], and here they were labeled as “SYN” as they developed a synthetic phenotype. Samples in which calcification was induced were labeled as “CAL” (details provided in the following section). Cells intended for immunofluorescence processing were cultured on glass coverslips.

### 2.2. Sample Processing for Transmission Electron Microscopy (TEM)

Confluent VSMCs, with or without 7 days of treatment with calcifying medium, were detached with trypsin and centrifuged at 300× *g* for 5 min. VSMC pellets were fixed in 2.5% glutaraldehyde in 0.1 M cacodylate buffer, pH 7.2, for 2 h at 4C, washed in the same buffer and post-fixed in osmium tetroxide in 0.1 M cacodylate buffer for 1 h at room temperature. After dehydration in a graduated ethanol series, the samples were transferred into propylene oxide for 15 min, embedded in Epon-Araldite, and finally polymerized at 60 °C. Ultrathin sections (70–90 nm), obtained with a Reichert-Jung Ultracut E (Reichert-yung, Wien, Austria) equipped with a diamond knife, were collected on 200-mesh formvar/carbon-coated copper grids, double-stained with uranyl acetate and lead citrate, and examined with a Jeol 100 SX transmission electron microscope (Jeol, Tokyo, Japan) operating at 80 kV. Micrographs were obtained with an AMTXR80b Camera System (Advanced Microscopy Technique, Woburn, MA, USA).

### 2.3. Cellular Calcification Induction

Before each experiment, the calcifying medium was freshly prepared. A 100 mM stock solution of phosphates (NaH_2_PO_4_ and Na_2_HPO_4_ in distilled water) was prepared and filter-sterilized using a 0.2 µm filter. The stock solution was then added to the DMEM high glucose (plus antibiotic–antimycotic solution) to achieve a final concentration of 1.9 mM, as indicated in our previous papers [17,27,28]. To allow the calcification process to occur, VSMCs were left for 7 days before being harvested for subsequent analysis. Therefore, the results from such treated cells were compared with SYN samples only, as VSMCs cannot survive under serum-starving conditions for 7 days.

### 2.4. Cell Lysis and Intracellular Calcium Quantification

Cells were processed similarly to what was reported in our previous papers [17,27,28]. VSMCs were gently washed twice with PBS and then lysed by adding 200 µL of 0.6 M HCl per well for a 12-well plate format. The plate was then incubated for 1 h at 4 °C. After this incubation, cells were dislodged by vigorous pipetting and scraping with a pipette tip. The plate was subsequently transferred to a −20 °C freezer overnight to further enhance cell lysis. The following day, the samples were tested for calcium ion concentration using the Calcium Colorimetric Assay Kit (Merck, Darmstadt, Germany), which exploits a chromogenic complex formed between calcium ions and o-cresol phthalein. Six standard dilutions were freshly prepared, with a final volume of 50 µL. The standard dilutions were as follows: Blank (0.0 µg), 0.4 µg, 0.8 µg, 1.2 µg, 1.6 µg, and 2.0 µg. Enough volume was prepared to perform duplicates for each dilution. Fifty microliters of each sample were transferred to a 96-well transparent plate, including the diluted standards in the same plate. Ninety µL of chromogenic reagent was added to each well and gently mixed with the pipette, avoiding vigorous pipetting to prevent foam formation. Then, 60 µL of calcium assay buffer was added to each well, and the solution was gently mixed again. The plate was incubated for 10 min at room temperature and protected from light. Finally, absorbance was recorded at 575 nm.

### 2.5. Immunofluorescence Analysis

The samples were prepared for examination and analysis using laser scanning confocal microscopy (LSCM). To fix the cells, a 2% paraformaldehyde solution in phosphate-buffered saline (PBS) was applied for 30 min at 4 °C. Three successive washes with PBS without calcium and magnesium (Merck, Darmstadt, Germany) were conducted between the outlined steps, and all incubations were carried out at room temperature. The permeabilization step involved treating the cells with 0.1% Triton-X (Merck, Darmstadt, Germany) in PBS for 5 min, followed by blocking with a solution of 5% BSA and 0.1% Tween-20 (Merck, Darmstadt, Germany) in PBS for 1 h. Next, the cells were incubated in the dark for 30 min at room temperature, with Phalloidin Alexa Fluor 488 (Santa Cruz Biotechnology, Dallas, TX, USA) at a 1:1000 dilution in the blocking solution. Alternatively, samples were incubated overnight at 4 °C with the following primary antibodies: Rabbit Monoclonal Antibody (MAb) Alpha Smooth Muscle Actin (Thermo Fisher, Waltham, MA, USA) at a 1:200 dilution in the blocking solution and Mouse MAb Galectin 3 (Thermo Fisher, Waltham, MA, USA) at a 1:100 dilution in the blocking solution. For secondary antibodies, Goat anti-Mouse Alexa Fluor Plus 488 (Thermo Fisher, Waltham, MA, USA) at a 1:2000 dilution in blocking solution or Goat anti-Rabbit Alexa Fluor Plus 594 (Thermo Fisher, Waltham, MA, USA) at a 1:2000 dilution in blocking solution was used, and samples were incubated for 2 h shielded from light. Following the staining steps, coverslips were mounted on glass slides using Fluoroshield™ with DAPI (Merck, Darmstadt, Germany). Samples were examined, and images were acquired using a laser scanning confocal microscopy system. All images were taken using a Leica TCS SP8 (Leica Microsystems, Mannheim, Germany) with the XYZ scan mode and an HC PL APO CS2 40x with a 1.30 oil objective. Representative confocal images were captured in 3D at a resolution of 2048 × 2048 pixels.

### 2.6. Total RNA Extraction, Quantification, and Quality Assessment, cDNA Synthesis

Cell pellets were collected by thoroughly scraping with pipette tips in PBS for later gene expression analysis. Total RNA was extracted using the PureLink™ RNA Mini Kit (Thermo Fisher, Waltham, MA, USA) according to the manufacturer’s instructions. To enhance the efficiency and purity of the RNA, on-column DNase treatment was performed. The RNA concentration and purity were measured using a spectrophotometer (NanoDrop™ Lite Spectrophotometer, Thermo Fisher, Waltham, MA, USA), and the samples were stored at −80 °C until further use. Complementary DNA (cDNA) was synthesized from the isolated total RNA (250 ng) using the SuperScript™ IV First-Strand Synthesis System (Thermo Fisher, Waltham, MA, USA), following the manufacturer’s guidelines. No-Reverse Transcriptase (NRT) samples were included in the reaction to ensure accuracy. If not used immediately, samples were stored at −20 °C.

### 2.7. Real-Time qPCR Analysis

The cDNA from each sample was diluted with DEPC-treated water to achieve a final concentration of 25 ng/µL per well and was loaded into a reaction plate in triplicates. Controls, including no-template controls (NTC) and no-reverse transcriptase (NRT), were included to check for contamination. Gene expression analysis was carried out using a SYBR™ Green-based assay (Thermo Fisher, Waltham, MA, USA), following the manufacturer’s guidelines. Primers for both forward and reverse directions were designed using Primer-BLAST (http://www.ncbi.nlm.nih.gov/tools/primer-blast accessed on 19 September 2024) and obtained from Sigma-Aldrich. The comprehensive list of primers used can be found in Table 1. The real-time PCR amplification and analysis were performed using a Bio-Rad CFX96™ Real-Time System (Bio-Rad Laboratories Inc., Hercules, CA, USA). The PCR cycling protocol is detailed in Table 1. After the amplification process, a melting curve analysis was conducted to confirm the purity of the amplicon. For the analysis following qPCR, Glyceraldehyde-3-Phosphate Dehydrogenase (GAPDH) was used for normalization, and relative quantification was determined using the 2^−ΔΔCt^ method.

### 2.8. Statistical Analysis

Unpaired *t*-test or ANOVA tests, either one-way or two-way as applicable, were performed, followed by Fisher’s LSD or Dunnett’s multiple comparison tests when necessary to compare the tested samples with the control untreated VSMCs. Results are expressed as the mean ± standard deviation (SD), with significance at *p*  <  0.05. Data were analyzed using GraphPad Prism version 9 (GraphPad9 Software, Boston, MA, USA). The data shown represent the averaged results of n = 3 independent experiments.

## 3. Results and Discussion

### 3.1. Confocal Imaging Reveals Morphological Changes During VSMCs Phenotypic Switch

**Figure 1:**
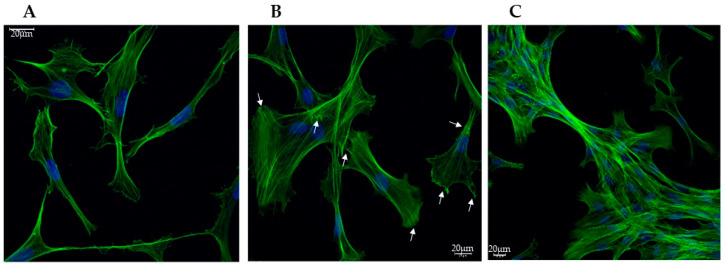
Representative confocal microscopy images showing the morphological change in VSMCs during the phenotypic switch. (**A**) Contractile (CON); (**B**) Synthetic (SYN), arrows point to focal adhesions; (**C**) Calcified (CAL) states. Fixed cells were stained with Phalloidin Alexa Fluor 488 (green) and DAPI (blue) to visualize nuclei. Scale bars indicate 20 µm.

VSMCs cultured in a standard medium with 10% FBS migrate and proliferate, showing a synthetic (SYN) phenotype [26]. When cultured in a medium deprived of serum for at least 3 days, they acquire the contractile (CON) quiescent phenotype. The green, fluorescent stain with Phalloidin Alexa Fluor 488 was used to evidence the overall morphology of the cells, enabling the visualization of the global structural changes and remodeling of the actin cytoskeleton that occurs during this phenotypic switch. As shown in Figure 1, the changes evidenced by the expression of phalloidin were associated with a morphological shift from spindle-shaped (Figure 1A), typical of the contractile phenotype, to fan-shaped forms, with many focal adhesions, characteristic of moving cells, thus of the synthetic phenotype (Figure 1B). When cells were treated with a calcifying medium, their general morphology was similar to the SYN ones, and they seemed to coalesce, forming tight bundles (Figure 1C).

### 3.2. Transmission Electron Microscopy Images Allow Us to Look Deeply Inside Vascular Smooth Cells

**Figure 2:**
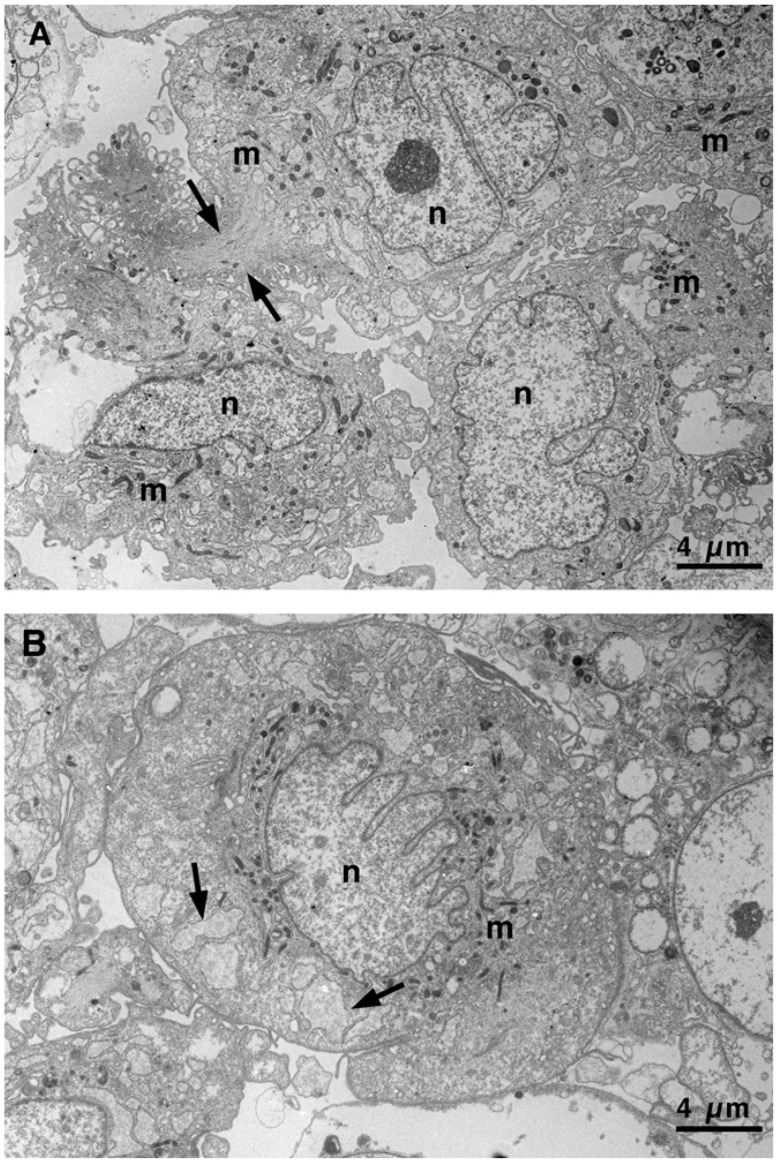
Transmission electron microscopy of VSMCs. (**A**) Representative image of CON VSMCs showing a large oblong nucleus, numerous mitochondria, and a bundle of myofibrils (arrows) in the cytoplasm. (**B**) Representative image of an SYN VSMC with a lobed nucleus and the cytoplasm displaying few scattered mitochondria and enlarged rough endoplasmic reticulum (RER) cisternae (arrow). m, mitochondrion; n, nucleus. Scale bar, 4 μm.

VSMCs were also processed for transmission electron microscopy. During the morphological shift from the CON (Figure 2A) to SYN (Figure 2B) phenotype, cells became bigger (see scale bar), and nuclei acquired a characteristic lobed, irregular shape. Mitochondria were more numerous inside CON VSMCs, as expected in cells that need energy for contraction.

**Figure 3:**
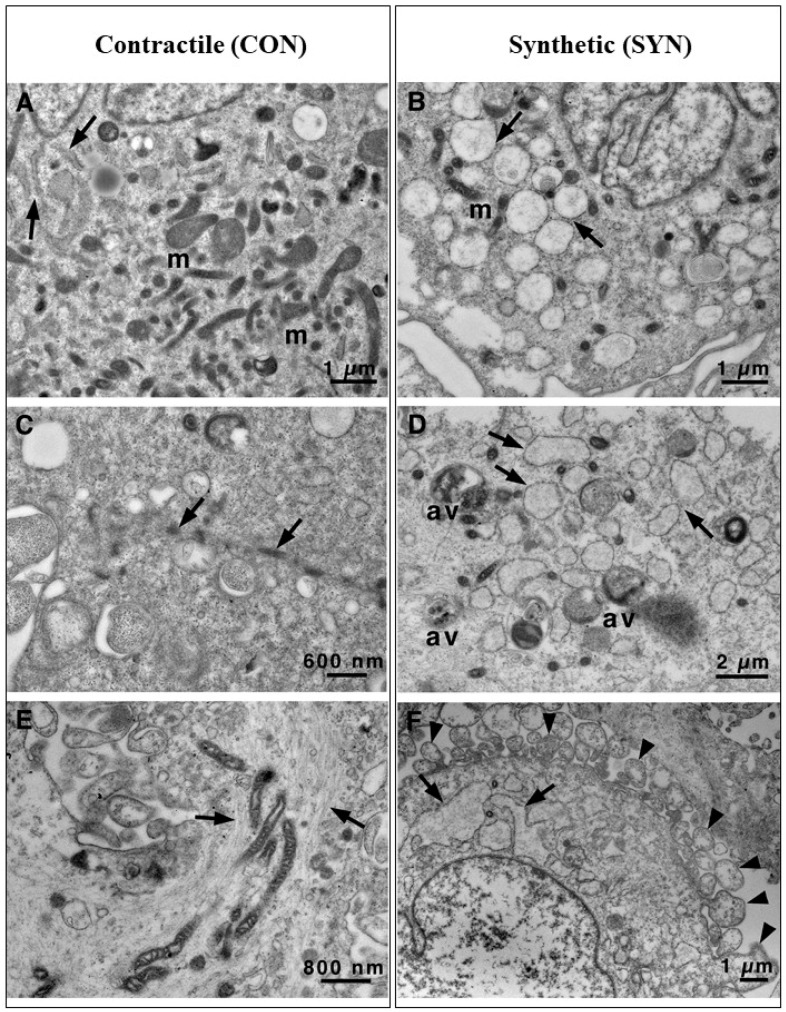
Transmission electron microscopy of VSMCs. Ultrastructural details of CON (**A**,**C**,**E**) vs. SYN (**B**,**D**,**F**) VMSC cytoplasm. A large number of mitochondria and regular cisternae of RER (arrow) are present in CON cells (**A**) concerning SYN cells (**B**), where scanty mitochondria and large roundish cisternae of RER (arrow) are visible. Bundles of myofibrils, sometimes mixed with mitochondria ((**C**,**E**) arrows), are present in CON cells. At the same time, they are scarce or absent in SYN cells where autophagic vacuoles and enlarged RER cisternae (arrow) are the main features (**B**,**D**,**F**). In SYN cells, the plasma membrane shows numerous pseudopod-like structures ((**F**) arrowheads). av, autophagic vacuole; m, mitochondrion; RER, rough endoplasmic reticulum. Scale bars: (**A**,**B**,**F**) 1 μm; (**C**) 600 nm; (**D**) 2 μm; (**E**) 800 nm.

The huge number of mitochondria in CON VSMCs is also highlighted in Figure 3A. The endoplasmic reticulum was highly represented in SYN VSMCs and composed of enlarged roundish cisternae, while in CON cells, it had a more standard aspect, composed of narrow cisternae (Figure 3A). Bundles of myofibrils with dense bodies were the main features of CON VSMCs (Figure 3C,E); moreover, myofibrils were associated with mitochondria (Figure 3E). In addition, autophagic vacuoles and membrane cell protrusions (see arrows), likely pseudopods that characterize the cell surface (Figure 3F), were peculiar hallmarks for SYN VSMCs (Figure 3D). The presence of pseudopods clearly demonstrated that SYN VSMCs were migrating cells. Basal autophagy is an essential mechanism in maintaining cellular homeostasis in all cell types, allowing the recycling and clearance of damaged proteins and organelles. Deregulation of one or more steps in this process (autophagy activation, autophagosome elongation and maturation, and autolysosomal content degradation) can make a decisive contribution to the pathogenesis of several diseases, including cardiovascular pathologies [10,11,12]. Recently, several studies showed that autophagy occurs in atherosclerosis and, in VSMCs, seems to be a potentially vital mechanism to maintain the stability of advanced plaques, exerting a positive effect [29]. On the other hand, an excessive autophagy induced the phenotypic transition to the de-differentiated (i.e., pathological) state, promoting VSMC proliferation and vascular remodeling that contributed to the pathogenesis of atherosclerosis [30,31].

**Figure 4:**
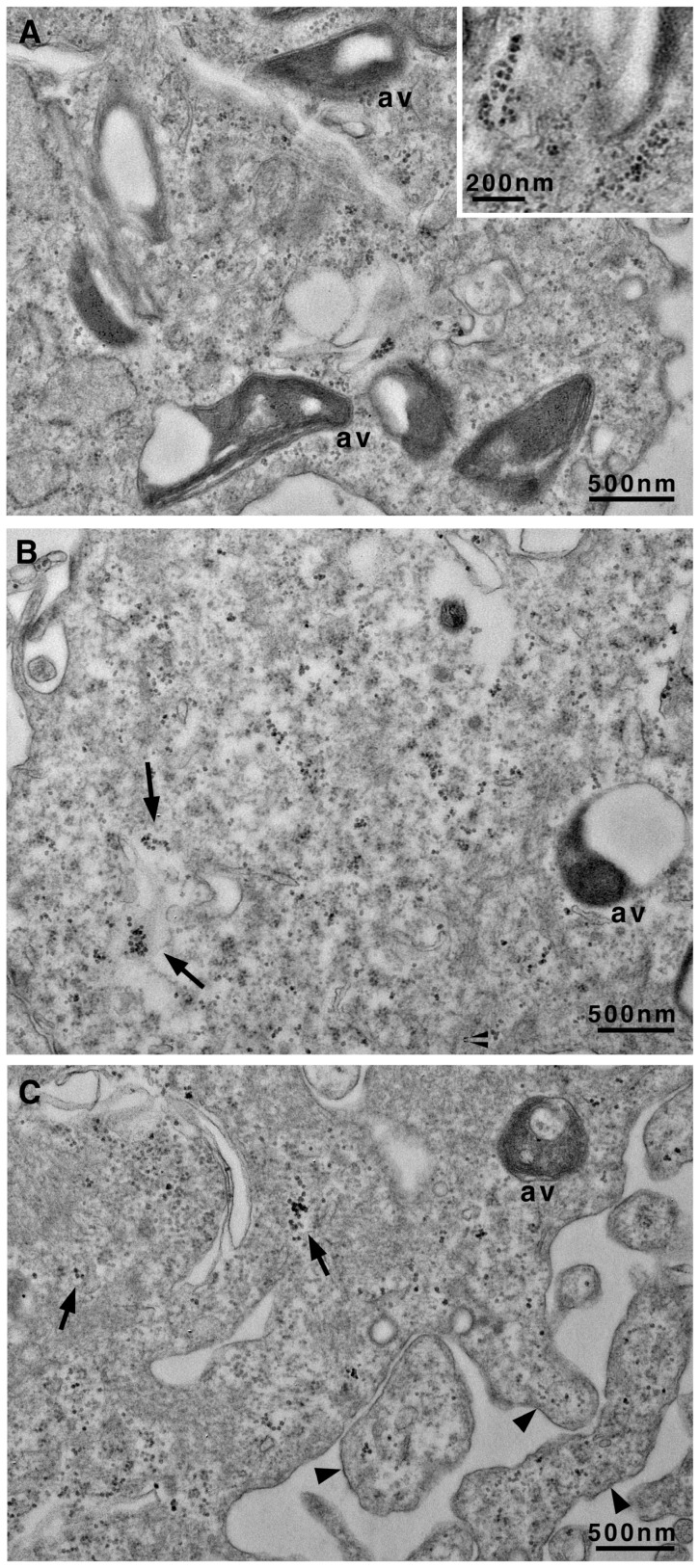
Transmission electron microscopy of VSMCs cultured in calcifying medium for 7 days. Representative images showing scattered calcium microcrystals of different sizes in the cytoplasm ((**A**) with insert and (**B**,**C**)). In (**B**,**C**), microcrystals are also evident inside vesicles (arrow) and in the surface protrusions (arrowhead) of the plasma membrane (**C**). av, autophagic vacuoles. Scale bars, 500 nm; insert, 200 nm.

In more advanced stages of atherosclerosis, VSMCs assumed a chondrocyte-like phenotype, an essential event in fibroatheroma formation. This shift is associated with the initiation of calcification and the formation of microcalcifications in the necrotic core of the atherosclerotic plaque [32]. In our in vitro model, VSMCs treated with the calcifying medium for at least 7 days maintained specific characters of the SYN phenotype and, in particular, autophagic vacuoles remained abundant (Figure 4), and endoplasmic cisternae were still enlarged (Figure 4A). The onset of calcification was demonstrated by the presence of microcrystals scattered throughout the cytoplasm (arrows in Figure 4B,C) but also in vacuoles and cell protrusions (arrowheads in Figure 4C).

**Figure 5:**
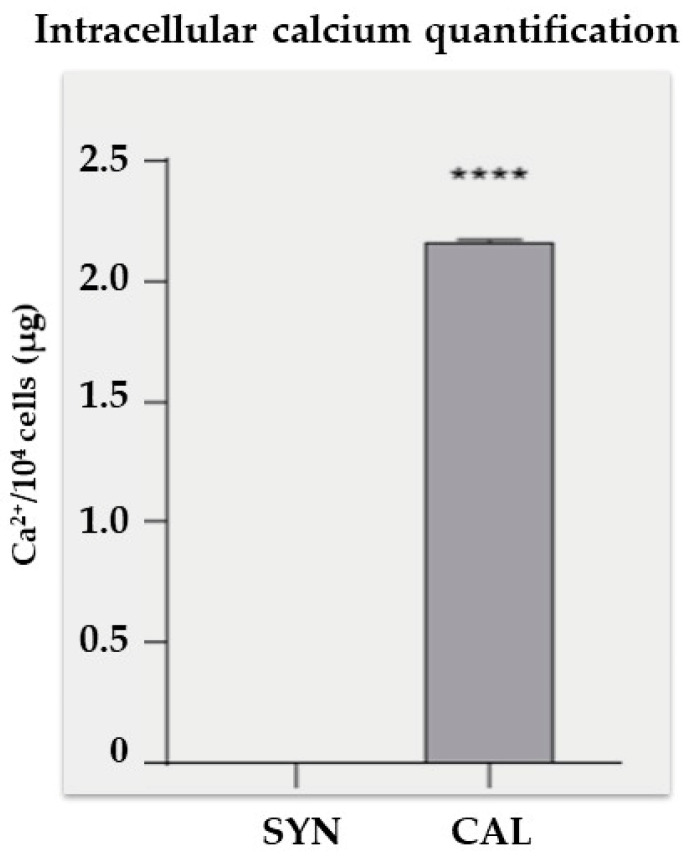
Intracellular calcium quantification. VSMCs were exposed to a phosphate mixture for 7 days, then lysed, and intracellular calcium levels were measured. Statistical analysis was conducted using a *t*-test; the bars indicate the mean ± SD. **** *p* < 0.0001. The data shown represent the averaged results of n = 3 independent experiments.

Intracellular calcium analysis performed with a colorimetric assay revealed a significant increase in calcium levels in the CAL group compared to the SYN group (Figure 5). The CAL group showed an average calcium concentration of approximately 2.1 µg per 10^4^ cells. In contrast, the SYN group exhibited negligible calcium content, highlighting the impact of the treatment on cellular calcium accumulation.

**Figure 6:**
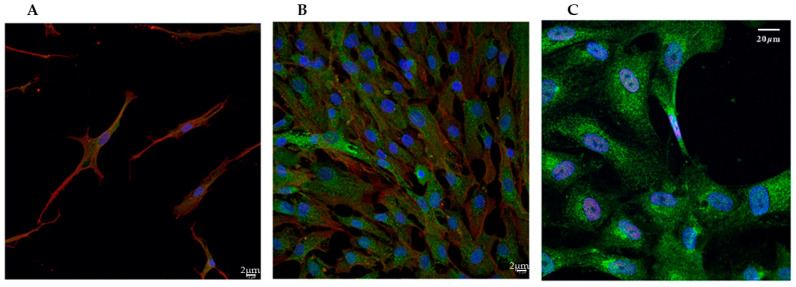
Evaluation of activation markers in VSMCs. Confocal microscopy images illustrate VSMCs cultured without serum (CON, (**A**)), with serum (SYN, (**B**)), and with calcifying media (CAL, (**C**)) to define the different phenotypes. In (**A**,**B**), the cells were fixed and stained with antibodies against α-smooth muscle actin (shown in red in the cytoplasm), galectin-3 (shown in green in the cytoplasm), in C cells stained with antibodies against RUNX-2 (shown in red in the nucleus) and galectin-3 (shown in green in the cytoplasm). DAPI was used for the nuclear visualization (blue). Scale bars: (**A**,**B**) 2 μm; (**C**) 20 µm.

**Figure 7:**
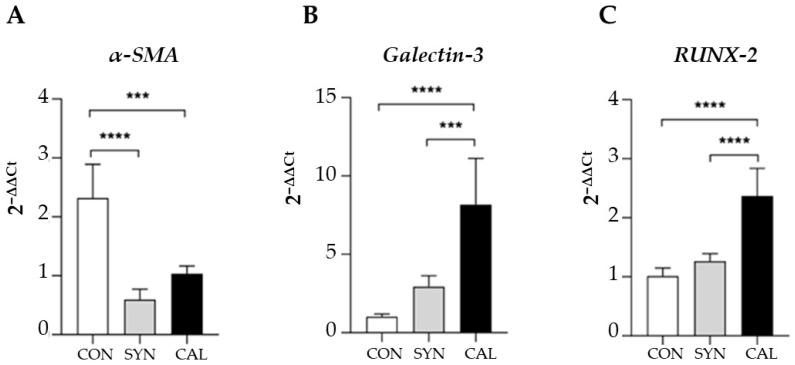
The relative expression levels of *α-SMA* (**A**), *galectin-3* (**B**), and *RUNX-2* (**C**) in VSMCs exposed to the different inducing media were assessed. mRNA expression levels were determined using real-time PCR, with normalization to *GAPDH*. The data presented are means ± SD. Statistical analyses were conducted using one-way ANOVA with Tukey’s post hoc test, with significance levels indicated as *** *p* < 0.001, **** *p* < 0.0001. The data shown represent the averaged results of n = 3 independent experiments.

We also investigated the phenotypic changes by exploiting immunofluorescence confocal microscopy to analyze the expression of specific contractile and synthetic biomarkers such as anti-α-SMA and anti-galectin-3, a marker for the SYN phenotype (Figure 6) [20]. As anticipated, cells cultured without serum (CON) showed high α-SMA expression (Figure 6A). In contrast, compared to CON, galectin-3 expression increased significantly in both SYN (Figure 6B) and CAL (Figure 6C) groups. RUNX-2 expression, an indicator of the synthetic-osteoblastic phenotype [5,14,15], was elevated in phosphate-treated samples compared to the CON and SYN phenotypes. Real-time PCR analysis (Figure 7) confirmed the morphological results since *α-SMA* expression decreased in the SYN and calcification-induced group (Figure 7A). *Galectin-3* (Figure 7B) showed reduced signal intensity in the CON group, whereas the calcification-exposed group exhibited a statistically significant increase. *RUNX-2* expressions significantly increased in CAL groups with respect to CON and SYN (Figure 7C), demonstrating distinct pathological phenotypes.

## 4. Conclusions

VSMCs are the predominant cell type within the arterial wall, where they exist in a quiescent, contractile state and exert the principal function of regulating vascular tone. Although VSMCS are mature, differentiated cells, they nevertheless retain phenotypic plasticity and can de-differentiate into a proliferative, synthetic state. This phenotypic modulation is associated with developmental and disease-associated vessel remodeling. Following environmental and hormonal stimuli (i.e., the calcium–phosphorus balance, the amounts of calcification inhibitors, mitochondrial dysfunction, endoplasmic reticulum stress), synthetic VSMCs can transit to osteoblast and chondrocyte-like phenotypes [33,34]. This shift is associated with the appearance of calcifying vesicles and the formation of microcalcifications in the necrotic core of the atherosclerotic plaque [32]. When VSMCs are isolated from arteries and cultured in vitro with bovine serum, they abandon their quiescent state and divide and migrate, becoming synthetic [26]. This activation is due to factors and hormones present in the serum. However, these cells can survive in a serum-deprived medium and return to a quiescent state characterized by the spindle-shaped morphology typical of the contractile phenotype. Moreover, it is possible to induce calcification of synthetic/activated VSMCs by culturing the cells in a high phosphate medium for at least 7 days [17,27,28]. Exploiting this in vitro model, our study confirmed the morphological VSMC plasticity and phenotypic switching. It provided substantial insights into the ultrastructural features of the cells during the differentiation from the contractile, to synthetic, and eventually to calcified phenotype. The study’s main findings using transmission electron microscopy and confocal immunofluorescence analyses included remodeling of the cytoskeleton, enlarged RER cisternae, increased autophagic vacuoles in synthetic VSMCs, and calcium microcrystal deposition in calcified cells. Real-time PCR further validated these changes, showing the increase in *galectin-3* and *RUNX-2*, and the reduction in *α-SMA* in synthetic and calcified VSMCs. Phenotypic modulation of VSMCs is associated with the expression of several genes and variants, but the changes at morphological and ultrastructural levels due to this modulation have not been thoroughly investigated [35,36]. Gaining better insights into the cell characteristics and components is an essential step in comprehending the role of VSMCs during atherosclerosis onset and progression. It can also help develop targeted therapies to slow plaque formation and mitigate vascular calcification.

### Limitations

Although 2D models based on VSMCs are widely used for pathophysiological investigations, they have several limitations [37,38]. Firstly, the stiffness of tissue culture support differs from the stiffness of the vessel wall, influencing the VSMC de-differentiation and leading to an altered ECM secretion [39]. Regarding the phenotype, which is the topic of our study, VSMCs in vitro have a synthetic phenotype due to the presence of FBS in the culture medium [26]. This experimental condition makes it difficult to maintain their contractile phenotype, which can be achieved by starvation necessarily reducing cell culture timings due to the absence of FBS. In addition, for TEM analysis, it is necessary to detach the cells from the substrate, and this can lead to cell rearrangements that could affect the ultrastructural analysis. Like all static 2D models developed to reproduce vascular tissue, our model has the limitation of not mimicking the hemodynamic flow conditions that contribute to the rearrangement of cell morphology. Recently, our research group developed a dynamic vascular tissue model that better mimics the in vivo condition; however, an ultrastructural morphological analysis of VSMCs is not possible in that experimental context due to the impossibility of recovering them as cell pellets [27,28].

## Figures and Tables

**Table 1:** List of primers used in this study.

Gene	Primer Sequence (5′–3′)
*GAPDH*	FWD: ACATCGCTCAGACACCATGG REV: GACGGTGCCATGGAATTTGC
*RUNX2*	FWD: GATTCTTAACCAACCAGCCTTACC REV: AGTGATGTCATTCTGCTCCTCTAA
*α-SMA*	FWD: AGAGTTACGAGTTGCCTGATG REV: GATGAAGGATGGCTGGAACA
*Galectin-3*	FWD: GCCACTGATTGTGCCTTATT REV: CCGTGCCCAGAATTGTTAT

## Data Availability

The original contributions presented in the study are included in the article. Further inquiries can be directed to the corresponding author.

## Abbreviations

The following abbreviations are used in this manuscript:

α-SMA	Alpha-Smooth Muscle Actin
BSA	Bovine Serum Albumin
CAL	Calcified
CON	Contractile
DMEM	Dulbecco’s Modified Eagle Medium
FBS	Fetal Bovine Serum
GAPDH	Glyceraldehyde-3-Phosphate Dehydrogenase
HCASMCs	Human Coronary Artery Smooth Muscle Cells
LSCM	Scanning Confocal Microscopy
PBS	Phosphate-Buffered Saline
RER	Rough Endoplasmic Reticulum
RUNX-2	Runt-Related Transcription Factor 2
SYN	Synthetic
TEM	Transmission Electron Microscopy
VSMCs	Vascular Smooth Muscle Cells
